# The challenge of multidrug resistance in hospitalized pediatric patients with urinary tract infections

**DOI:** 10.3389/fcimb.2025.1570405

**Published:** 2025-07-01

**Authors:** Zeinab El Zein, Celina F. Boutros, Marwa El Masri, Elsy El Tawil, Maher Sraj, Yara Salameh, Sarah Ghadban, Rim Salameh, Silma El Baasiri, Amani Haddara, Mayse Nasser, Shady Tabbara, Sarah Khafaja, Rawan Korman, Soha Ghanem, Dany Al Hamod, Ghassan S. Dbaibo

**Affiliations:** ^1^ Center for Infectious Diseases Research, WHO Collaborating Center, American University of Beirut, Beirut, Lebanon; ^2^ Division of Pediatric Infectious Diseases, Department of Pediatrics and Adolescent Medicine, American University of Beirut Medical Center, Beirut, Lebanon; ^3^ Department of Pediatrics, Saint George Hospital University Medical Center, Beirut, Lebanon; ^4^ Faculty of Medicine, University of Balamand, Mount Lebanon, Lebanon; ^5^ Department of Pediatrics and Adolescent Medicine, American University of Beirut Medical Center, Beirut, Lebanon

**Keywords:** urinary tract infection, children, extended-spectrum beta lactamase, multidrug resistant organisms, antimicrobial resistance, Lebanon

## Abstract

**Background:**

The choice of empirical treatment in pediatric urinary tract infections (UTIs) is increasingly complicated by the emergence of antibiotic resistance and the growing prevalence of multidrug-resistant organisms (MDROs). The aim of this study is to assess the resistance patterns of isolated uropathogens among children and adolescents hospitalized with UTIs in Lebanon; and determine the risk factors associated with MDRO-related UTIs over a 10-year period.

**Methods:**

A retrospective chart review was conducted at two tertiary medical centers in Beirut. Children and adolescents less than 18 years who were admitted, between January 1, 2011, and December 31, 2021, with the following ICD-10 codes: “urinary tract infection”, “cystitis” and/or “pyelonephritis “ were included. A case was excluded if the urine culture was polymicrobial or did not meet the definition of UTI we used. Univariate and multivariable logistic regression analyses were performed to identify risk factors for MDRO infections.

**Results:**

Among the 876 pediatric UTI cases included, 85% were above 2 months of age and 74.1% were females. 64.5% of 644 *Escherichia coli* and 61.9% of 114 *Klebsiella* spp. isolates met international MDR criteria. After a period of fluctuation, the proportion of MDROs began to steadily increase starting 2019 eventually surpassing the 2011 percentage by nearly 10% in 2021 (67.9%, *p* = 0.248). Only 2.1% of MDR *E. coli* and 2.9% of MDR *Klebsiella* spp. were resistant to carbapenems. However, aminoglycoside resistance was high ranging between 28.3% and 48.6%. Children aged ≥ 5 years were nearly twice as likely to present with an MDR uropathogen compared to those < 5 years of age (*p* < 0.001). Only a history of leukemia (*p* = 0.010, AOR = 4.248, 95% CI [1.412–12.778]) and antibiotic use in the preceding 30 days (*p* = 0.012, AOR = 2.045, 95% CI [1.167–3.582]) were found as independent risk factors for UTIs caused by MDROs in multivariable logistic regression.

**Conclusion:**

This study highlights the increasing threat of MDROs among pediatric UTIs. Recent antibiotic use was strongly associated with MDRO infections highlighting the urgent need for effective antimicrobial stewardship, re-evaluation of empiric treatment guidelines, and strict abidance by infection control measures.

## Introduction

Febrile urinary tract infections (UTIs) are now recognized as the most common cause of serious bacterial illness in children under the age of two years, as the widespread implementation of conjugate vaccines targeting *Haemophilus influenzae* and *Streptococcus pneumoniae* has significantly lowered the incidence of bacteremia, complicated pneumonia, and meningitis (Esposito et al., 2022). The majority of UTIs in children are caused by Gram-negative organisms from the *Enterobacterales* family, primarily *Escherichia coli*, which accounts for 80–90% of cases, followed by *Klebsiella* spp., *Proteus* spp., and *Enterobacter* spp. Other uropathogens, though less common, include *Pseudomonas aeruginosa* and Gram-positive pathogens such as *Enterococcus* spp. and *Staphylococcus saprophyticus* (Simões et al., 2020).

Studies showed that the risk of irreversible damage to the renal parenchyma gradually increases with each febrile UTI (Shaikh et al., 2019). Therefore, it is essential to start the appropriate antibiotic therapy as soon as possible to avoid such complications. However, the choice of empirical treatment is increasingly complicated by the emergence of antibiotic resistance and the growing prevalence of multidrug-resistant organisms (MDROs).

In 2017, the World Health Organization (WHO) included *Klebsiella*, *E.coli*, *Serratia*, and *Proteus* among a list of 12 genera of Gram-negative bacteria that constitute a public health threat due to their resistance to multiple antibiotics (Bryce et al., 2022). An international definition of MDROs was not established until 2010, by the European Centre for Disease Prevention and Control (ECDC) and the Centers for Disease Control and Prevention (CDC); as organisms resistant to at least one antimicrobial in three or more antibiotic classes based on *in vitro* testing (Magiorakos et al., 2012).

In *Enterobacterales*, the most common mechanism of resistance is the production of beta lactamases such as extended-spectrum beta lactamases (ESBLs), AmpC cephalosporinases, and carbapenemases. ESBLs hold particular importance because their genes are located on plasmids, allowing them to transfer across different species of *Enterobacterales* of varying virulence or with pre-existing resistance profiles (Moxon and Paulus, 2016; Mahony et al., 2020). This explains how MDROs, initially limited to healthcare settings, have also spread to community environments.

The overall estimated pooled prevalence of pediatric UTIs caused by uropathogens expressing ESBLs is 14% (Flokas et al., 2016), reaching almost 50% in Eastern Mediterranean Region studies (Topaloglu et al., 2010; Albaramki et al., 2019). In Lebanon, a retrospective chart review conducted at the American University of Beirut Medical Center (AUBMC) and Makassed General Hospital (MGH) over a 10 year period, showed an alarming increase in the incidence of ESBL-producing UTIs in hospitalized children, from 8% in 2001 to 25% in 2011 (Hanna-Wakim et al., 2015).

Previous studies have shown that renal abnormalities, vesicoureteral reflux (VUR), malignancies, hospitalization within the previous 3 months, recurrent UTI, and recent antibiotic use are independent risk factors for UTI caused by ESBL-producing uropathogens (Flokas et al., 2016).

Options for the treatment of MDR bacterial infections are generally limited, and few antibiotics are approved for use in children. In developing countries outside the Organization for Economic Co-operation and Development (OECD), including Lebanon, many antibiotics are available over the counter without a prescription, contributing to higher rates of resistant strains in these regions (Bryce et al., 2016; Dandachi et al., 2019). Concurrently, understanding antimicrobial susceptibility in different populations is crucial for guiding empiric therapy decisions.

Given these challenges, this retrospective chart review aimed to analyze the epidemiology and characteristics of UTIs in hospitalized children and adolescents in Lebanon at two tertiary care centers: AUBMC and Saint Georges Hospital University Medical Center (SGHUMC) between 2011-2021. The goal of this study is to assess further development in resistance patterns of the isolated organisms, and to identify the risk factors associated with MDRO-related UTIs.

## Methods

### Study design

This study was a retrospective review of medical records conducted over a ten-year period from January 1, 2011, to December 31, 2021, at two tertiary medical centers in Beirut, AUBMC and SGHUMC.

### Population

The study population included children and adolescents aged 0 to 18 years who were admitted to AUBMC or SGHUMC. Subjects were identified through the medical records departments at AUBMC and SGHUMC using the following International Classification of Diseases (ICD-10) codes: “urinary tract infection: N39. 0,” “cystitis: N30,” and “pyelonephritis: N10 “.

The exclusion criteria were the following: 1) adults > 18 years of age, 2) urine culture: polymicrobial, and 3) not meeting the UTI definition used in the study.

### Definition of UTI

UTI was defined based on established guidelines from the American Academy of Pediatrics, the National Institute for Health and Clinical Excellence, the European Society for Pediatric Urology, and the European Association of Urology (Stein et al., 2015; Mattoo et al., 2021). A UTI was defined as significant bacteriuria of a clinically relevant uropathogen in a symptomatic patient, meeting one of the following criteria: recovery of ≥ 100,000 colony-forming units per milliliter (CFU/mL) of a single uropathogen from a clean catch specimen; recovery of ≥ 50,000 CFU/mL of a single uropathogen from a catheterized specimen; recovery of ≥ 10,000 CFU/mL from catheterized urine specimens in the presence of pyuria and fever; or the presence of any uropathogen from a suprapubic aspirate. The criterion of 10,000 CFU/mL from catheterized specimens was included to account for laboratory variability in reporting bacterial counts. Polymicrobial urine cultures were excluded.

### Antimicrobial sensitivity and characterization

The identification of organisms and their sensitivities to antibiotics was carried out as reported by the Clinical Microbiology Laboratory, a College of American Pathologists-accredited laboratory since 2004 (CLSI, 2023).

In the analysis, the antimicrobials of interest were classified into classes and grouped together as follows:

Penicillins + betalactamase inhibitors (amoxicillin-clavaulinc acid), third generation cephalosporins (cefixime, ceftazidime, and cefotaxime), fourth generation cephalosporins (cefepime), fluoroquinoles (ciprofloxacin and levofoxacin), carbapenems (imipenem), aminoglycosides (amikacin and gentamicin), antipseudomonal penicillins + betalactamase inhibitors (piperacillin- tazobactam).

### Ethical considerations

Ethical approval was obtained from the Institutional Review Boards (IRBs) of AUBMC and SGHUMC (BIO-2022-0016), in accordance with the World Medical Association’s *Declaration of Helsinki* (2013) (Association WM, 2013). A waiver of informed consent was granted by the IRBs, as the study involved retrospective review of medical records with no risk to the subjects. All data collected were coded, with patient identifiers removed. To ensure confidentiality, the data was completed electronically through a password-protected website, RedCap, by the study investigators then transcribed into a secure database. The data was accessible only to the IRB-authorized research team. The database was regularly updated and saved on password-protected computers at the Center for Infectious Diseases Research (CIDR) office. All data will be destroyed once the legal retention period has expired.

### Study procedures and data collection tool

After securing IRB approval at both medical centers, the medical records (paper medical charts or electronic health records) of the subjects were screened, retrieved, and reviewed. The case report form was completed electronically using RedCap by the study investigators then transcribed into a secure database. The data collected included the following sections: 1) socio-demographic factors (age, gender), 2) past medical and surgical history, previous use of antibiotics, previous hospitalizations, history of diarrhea or constipation, and toilet training status, 3) insertion of foreign bodies with dates of insertion and removal (central venous catheter, arterial line, endotracheal tube, urinary catheter), 4) relevant signs and symptoms caused by the acute infection, and 5) hospital course [dates of admission and discharge, admission and discharge diagnoses, Intensive Care Unit (ICU) admission requirement, duration of hospital stay and ICU stay, method of urine culture, laboratory results at the time of infection, antimicrobial sensitivities, imaging [ultrasound of kidneys, voiding cystourethrogram, dimercapto succinic acid scan (DMSA)], and outcome on discharge.

### Statistical analysis

Data analysis was conducted using IBM’s Statistical Package for the Social Sciences (SPSS), version 29.0 for Windows (IBM, Armonk, NY). Descriptive statistics were employed to summarize patient demographics and UTI characteristics, overall distribution of different organisms between MDR and non-MDR infections, across pediatric age groups and over the years. Continuous variables were reported as means with standard deviations, whereas categorical variables were presented as frequencies and percentages. Univariate and multivariable logistic regression analyses were performed to identify risk factors for MDRO. The goodness-of-fit statistic (p > 0.05) was used to assess the model’s fit to the data. Associations were evaluated using the unadjusted odds ratio (UOR) for univariate analysis and the adjusted odds ratio (AOR) for multivariable analysis, both with 95% confidence intervals (CI). A p-value of ≤ 0.05 was considered statistically significant.

## Results

### Patient demographics and characteristics

Out of a total of 876 pediatric UTI cases included in the analysis, 600 cases were enrolled from AUB-MC following the exclusion of 1,562 cases from an initial pool of 2,162 screened cases. Additionally, 276 cases were enrolled from SGHUMC. Most of the patients were above 2 months of age: 15.2% ≤2 months, 41.7% > 2months - ≤ 2 years, and 43.2% ≥ 3 years. A female predominance was noted among the total cohort, with 649 females (74.1%) compared to 227 males (25.9%). However, among those ≤2 months of age (N=133), a slight male predominance of 54% was detected. Out of 92 males with known circumcision status, 39.1% were circumcised.

### Causative organisms and MDR rate over study period

**Figure 1:**
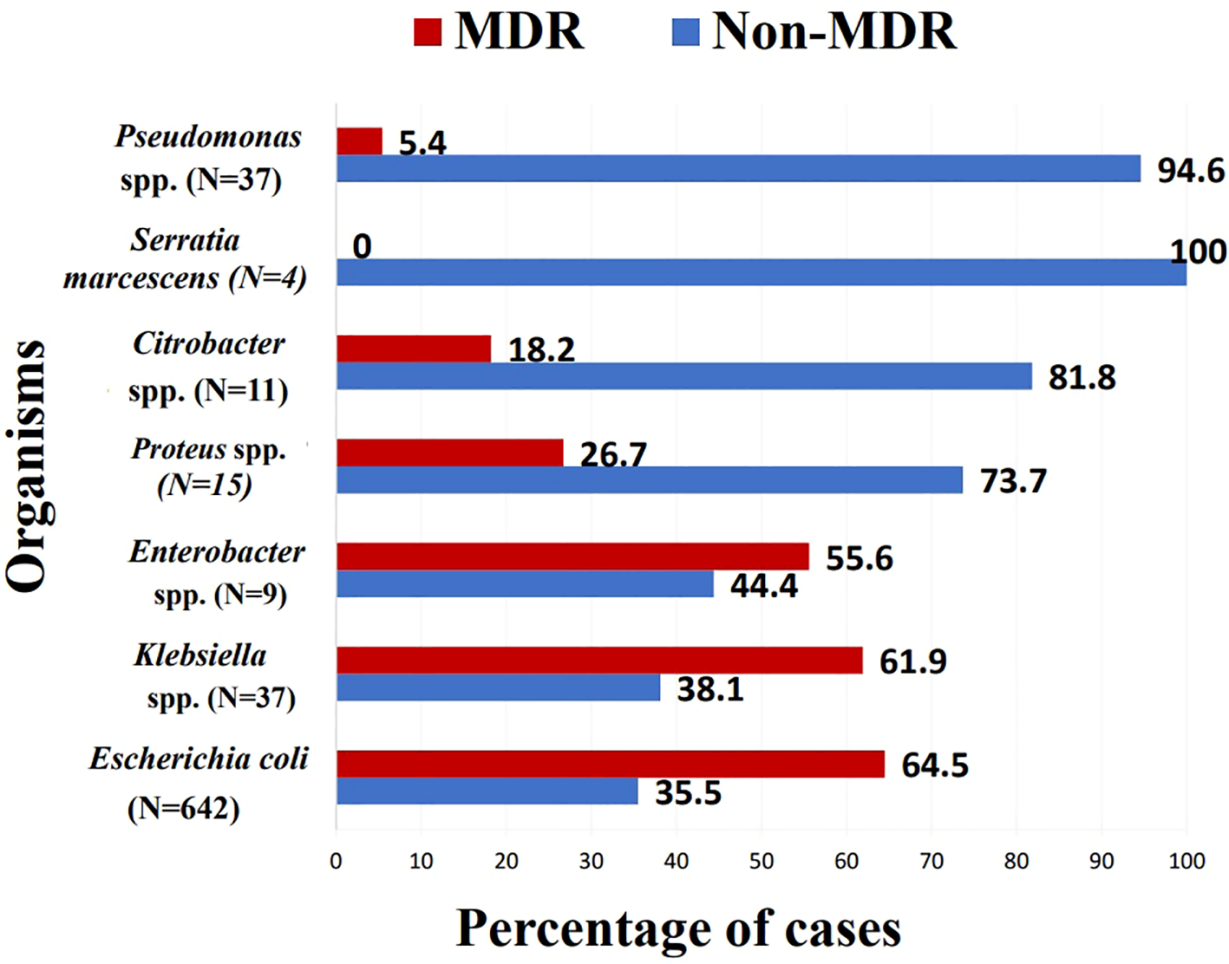
Percentage of multi drug resistant isolates within each species. MDR, multi-drug resistant; Spp., species.

The primary uropathogen encountered was *Escherichia coli* (73.9%), followed by Klebsiella spp. (13.1%) and Pseudomonas spp. (4.2%). Other gram-negative bacterial species such as Proteus, Citrobacter, and Enterobacter accounted for an additional 4% collectively. Gram-positive organisms were much less common, with Enterococcus spp. representing 2.5% and Staphylococcus spp. comprising 1% (**Supplementary Figure S1**). *E. coli* and Klebsiella spp. were more likely to exhibit multidrug resistance, where 64.5% of *E. coli* (*p* = 0.008, OR =8.171, 95% CI = [1.751 - 38.140]) and 61.9% of Klebsiella spp. (*p* =0.013, OR =7.326, 95% CI = [1.511 - 35.513]) isolates met international MDR criteria (Magiorakos et al., 2012) (**Figure 1**).

**Figure 2:**
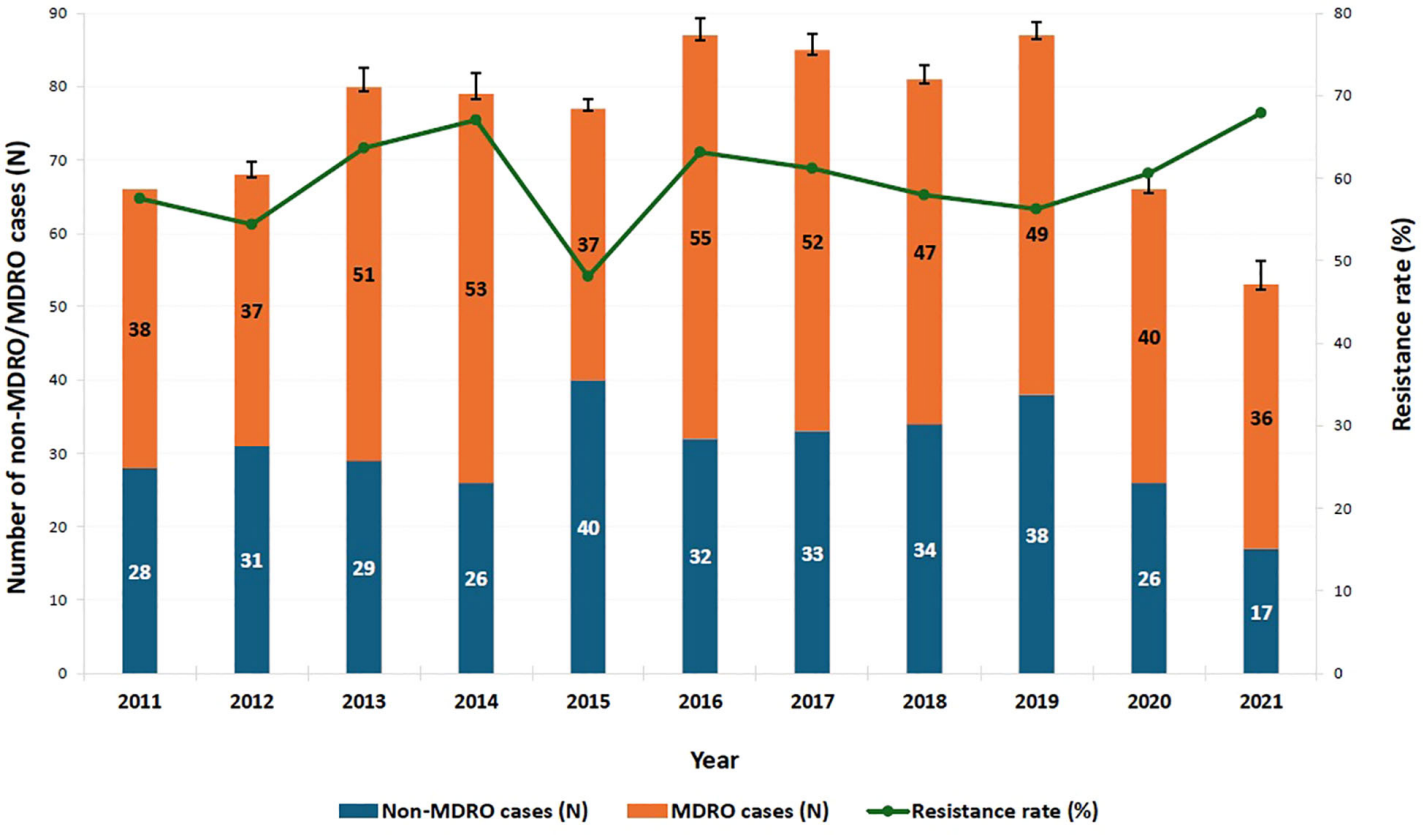
Trend of multi-drug resistance among identified pathogens over the study period. MDRO: multi-drug-resistant organism. A binary logistic regression analysis was performed to compare the proportion of MDROs across the years from 2011 to 2021, with 2011 serving as the reference year. After a period of fluctuation, the proportion of MDROs began to steadily increase starting in 2019, eventually surpassing the 2011 percentage by nearly 10% in 2021; however, this increase was not statistically significant (67.9%, p = 0.248, OR = 1.560, 95% CI = [0.733–3.322]). The y-axis represents the number of non-MDRO and MDRO cases. Error bars indicate 95% confidence intervals.

MDR rate over the study period ranged from 48.1% to 67.9%. Between 2011 and 2014, resistance rates fluctuated between 54.4% and 67%, with a noticeable decrease to 48.1% in 2015; though not statistically significant compared to 2011 (*p* = 0.256). Starting in 2019, the proportion of MDROs began to steadily increase, eventually surpassing the 2011 percentage by nearly 10% in 2021 (67.9%, *p* = 0.248) (**Figure 2**).

Among the total *E. coli* and Klebsiella spp. isolates, resistance to common oral antibiotics used in Lebanon for treatment of cystitis and pyelonephritis -penicillin + beta-lactamase inhibitors, third-generation cephalosporins, and folate pathway inhibitors- ranged from 30% to 50% (**Supplementary Figure S2**).

**Figure 3:**
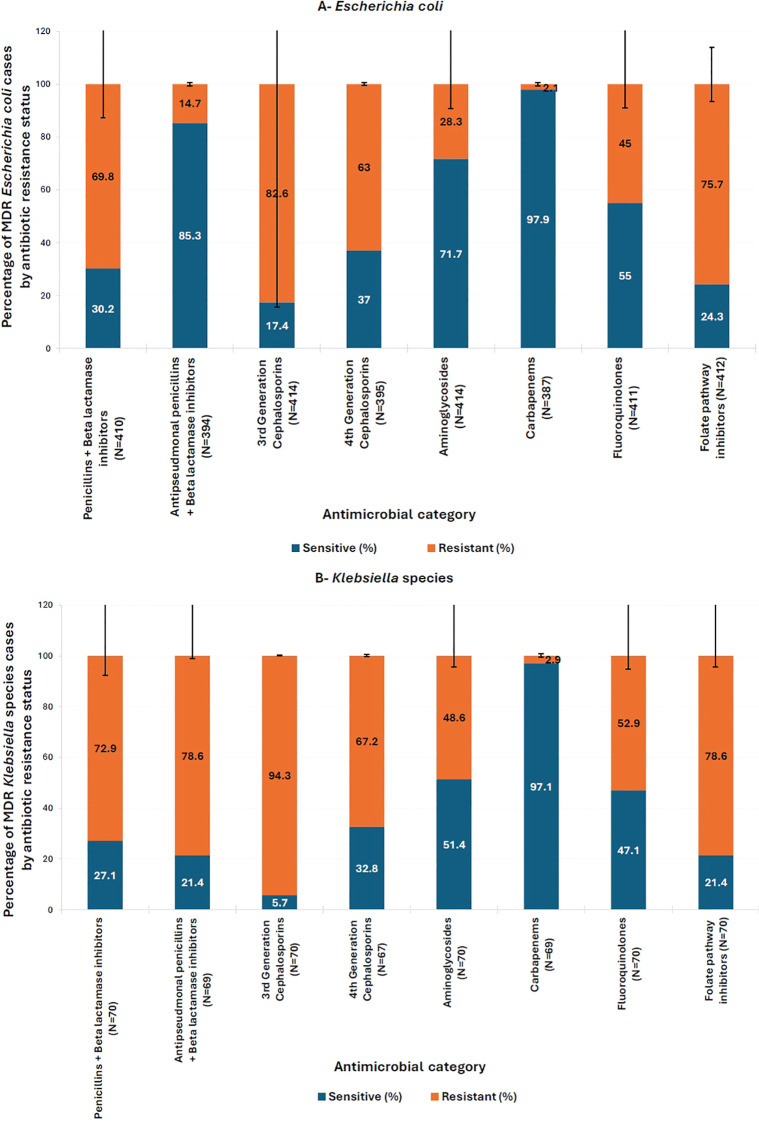
Distribution of resistance to different antimicrobial classes within multi-drug resistant (MDR) isolates of **(A)** *E*. *coli* and **(B)** *Klebsiella* spp. Antimicrobial Categories: Penicillins + beta-lactamase inhibitors (amoxicillin-clavulanic acid), antipseudomonal penicillins + betalactamase inhibitors (piperacillin-tazobactam), third generation cephalosporins (cefixime, ceftazidime, and cefotaxime), fourth generation cephalosporins (cefepime), folate pathway inhibitors (trimethoprim/sulfamethoxazole), fluoroquinolones (ciprofloxacin and levofloxacin), carbapenems (imipenem), aminoglycosides (amikacin and gentamicin). Pearson’s Chi-square test or Fisher’s exact test (for subgroups with fewer than five cases) was used to compare the percentage of multidrug-resistant (*Escherichia coli* and *Klebsiella* spp.) cases between antibiotic-sensitive and -resistant groups. All comparisons were statistically significant (p < 0.05), except for Carbapenems (p > 0.05). The y-axis represents the percentage of MDR *E*. *coli* and *Klebsiella* spp. cases by antibiotic resistance status. Error bars indicate 95% confidence intervals.

As for parenteral antibiotics frequently used for MDR infections, only 2.1% of MDR *E. coli* and 2.9% of MDR *Klebsiella* spp. were resistant to carbapenems, the only class of antibiotics for which MDR isolates were not significantly more resistant (p > 0.05). However, aminoglycoside resistance was high, particularly MDR *Klebsiella* spp. (48.6%), and to a lesser extent MDR *E. coli* (28.3%) (**Figure 3**).

### Trend of antibiotic resistance over time

**Figure 4:**
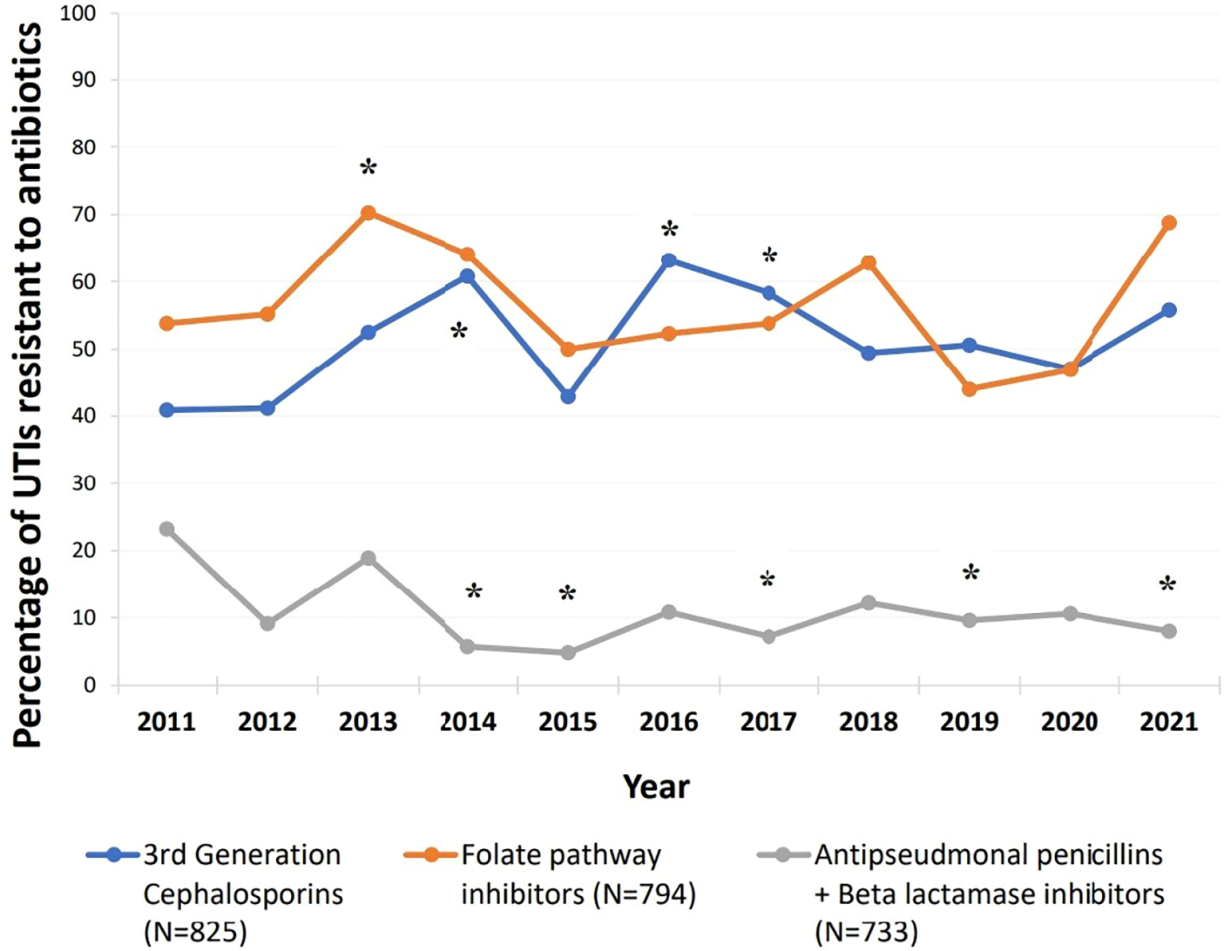
Trends of resistance to third generation cephalosporins, folate pathway inhibitors, and antipseudomonal penicillins + beta lactamase inhibitors between 2011-2021. Third generation cephalosporins (cefixime, ceftazidime, and cefotaxime), folate pathway inhibitors (trimethoprim/sulfamethoxazole), antipseudomonal penicillins + betalactamase inhibitors (piperacillin-tazobactam). *Compared to 2011: Resistance to third generation cephalosporins significantly increased in 2014: p=0.018, 2.237 [1.148- 4.357], 2016: p=0.007, 2.483 [1.288- 4.785], and 2017: p=0.035, 2.022 [1.051- 3.893]. Resistance to folate pathway inhibitors significantly increased in 2013: p=0.047, 2.026 [1.009- 4.069].Resistance to antipseudomonal penicillins + Beta lactamase inhibitors significantly decreased in 2014: p=0.008,0.2 [0.061- 0.655], 2015: p=0.008, 0.168 [0.045-0.627], 2017:p=0.016, 0.258 [0.086- 0.777], 2019: p=0.033,0.353 [0.135- 0.919], and 2021:p=0.041, 0.288 [0.087- 0.950].

Compared to 2011, resistance trends over the study period showed significant changes in certain antibiotic classes (*p* < 0.05) (**Figure 4**). Resistance to third-generation cephalosporins consistently increased from 40.9% in 2011, with particularly notable rises in 2014 (60.8%, *p* = 0.018), 2016 (63.2%, *p* = 0.007), and 2017 (58.3%, *p* = 0.035). For folate pathway inhibitors, resistance significantly increased by nearly 16% in 2013 (70.3%) compared to 2011 (53.8%, *p* = 0.047); then decreased over subsequent years to levels below those of 2011. However, a marked increase was again observed, from 46.9% in 2020 to 68.8% in 2021. In contrast, resistance to antipseudomonal penicillins combined with beta-lactamase inhibitors significantly decreased over the years, from 23.2% in 2011 to just 8% in 2021 (*p* = 0.041).

### Extended-Spectrum Beta-Lactamase producers

Over the study period, 44.6% of total *E. coli* and *Klebsiella* spp. isolates were categorized as ESBL producers. A higher proportion was observed among MDR isolates: 66.6% of MDR *E. coli* and 84.3% of MDR *Klebsiella* spp. The proportion of ESBL producers consistently exceeded the 2011 baseline level of 26.9% (**Supplementary Figure S3**). However, this increase was only statistically significant in 2014 (57.7%, *p* < 0.001), 2016 (52.9%, *p* = 0.001), and 2021 (45.1%, *p* = 0.041).

### Risk factors for MDR

Children aged ≥ 5 years were nearly twice as likely to present with an MDR uropathogen compared to those < 5 years of age (*p* < 0.001, OR = 1.884, 95% CI [1.383–2.567]) (**Table 1**). Univariate analysis identified other several significant factors significantly associated with UTIs caused by MDRO (*p* ≤ 0.05) (**Table 1**): stool toilet training, recurrent UTIs, underlying neurological diseases, immunosuppressive conditions, malignancies (including leukemia and solid tumors), chemotherapy, radiation therapy, recent non-genitourinary surgeries, antibiotic use within 30 days preceding admission, suppressive antibiotic therapy, hospitalization within the previous month, and the presence of a central venous catheter.

**Table 1:** Univariate analysis of potential risk factors associated with multi-drug-resistant organisms (MDROs).

Potential Risk Factors	Total	Non-MDRO	MDRO	p-value	UOR [95% CI]
Age					
***<5 years***	563/831 (67.7)	253/563 (44.9%)	310/563 (55.1%)	Ref	
***≥5 years***	268/831 (32.3)	81/268 (30.2%)	187/268 (69.8%)	**<0.001**	**1.884 [1.383- 2.567]**
Gender					
***Male***	203/831 (24.4)	92/334 (27.5)	111/497 (22.3)	Ref	
***Female***	628/831 (75.6)	242/334 (72.5)	386/497 (77.7)	0.087	
**Urine toilet training**	268/304 (88.2)	84/96 (87.5)	184/208 (88.5)	0.809	
**Stools toilet training**	271/508 (53.3)	84/190 (44.2)	187/318 (58.8)	**0.001**	**1.801 [1.253 - 2.590]**
**ATB use in the preceding 30 days before admission**	253/781 (32.4)	69/309 (22.3)	184/472 (39.0)	**<0.001**	**2.222 [1.605 - 3.077]**
*Penicillin*	34/253 (13.4)	15/69 (21.7)	19/184 (10.3)	**0.018**	**0.415 [0.197 - 0.872]**
*Third Generation Cephalosporins*	131/252 (52.0)	25/69 (36.2)	106/183 (57.9)	**0.003**	**2.423 [1.368 - 4.292]**
**Use of suppressive ATB**	144/820 (17.6)	41/333 (12.3)	103/487 (21.1)	**0.001**	**1.910 [1.290 - 2.829]**
**Previous hospitalization within 30 days before current admission**	187/780 (24.0)	52/304 (17.1)	135/476 (28.4)	**<0.001**	**1.919 [1.340 - 2.747]**
**VUR**	83/831 (10.0)	27/334 (8.1)	56/497 (11.3)	0.133	
**Recurrent UTI**	285/831 (34.3)	78/334 (23.4)	207/497 (41.6)	**<0.001**	**2.343 [1.718 - 3.194]**
**Neurological disease**	111/830 (13.4)	35/334 (10.5)	76/496 (15.3)	**0.044**	**1.546 [1.009 - 2.369]**
**Immunosuppression**	142/831 (17.1)	34/334 (10.2)	108/497 (21.7)	**<0.001**	**2.450 [1.620 - 3.705]**
**PID**	7/831 (0.8)	1/334 (0.3)	6/497 (1.2)	0.252*	
**Malignancy**	137/831 (16.5)	35/334 (10.5)	102/497 (20.5)	**<0.001**	**2.206 [1.461 - 3.332]**
**Leukemia**	68/831 (8.2)	12/334 (3.6)	56/497 (11.3)	**<0.001**	**3.407 [1.797 - 6.461]**
**Lymphoma**	2/831 (0.2)	0/334 (0.0)	2/497 (0.4)	0.519*	
**Solid tumors**	86/831 (10.3)	24/334 (7.2)	62/497 (12.5)	**0.014**	**1.841 [1.124 - 3.015]**
**Chemotherapy**	145/831 (17.4)	34/334 (10.2)	111/497 (22.3)	**<0.001**	**2.537 [1.679 - 3.834]**
**Radiation therapy**	33/831 (4.0)	6/334 (1.8)	27/497 (5.4)	**0.008**	**3.140 [1.282 - 7.692]**
**Non-GU surgery**	208/831 (25.0)	52/334 (15.6)	156/497 (31.4)	**<0.001**	**2.481 [1.746 - 3.525]**
Foreign bodies					
**CVC**	168/821 (20.5)	49/332 (14.8)	119/489 (24.3)	**0.001**	**1.858 [1.287 - 2.681]**
*Femoral CVC*	12/831 (1.4)	0/334 (0.0)	12/497 (2.4)	0.002*	**0.592 [0.559 - 0.627]**
*Jugular CVC*	19/831 (2.3)	5/334 (1.5)	14/497 (2.8)	0.212	
*Subclavian CVC*	3/831 (0.4)	0/334 (0.0)	3/497 (0.4)	0.278*	
*Polysite*	97/831 (11.7)	23/334 (6.9)	74/497 (14.9)	**0.001**	**2.366 [1.449 - 3.862]**
**Urinary catheter**	70/780 (9.0)	22/318 (6.9)	48/462 (10.4)	0.096	

GU, genitourinary; PID, primary immunodeficiency; *CVC, central venous* catheter.

Variables that did not show significance:

The use of any of the following antibiotics in the preceding 30 days before admission: first generation cephalosporins, second generation cephalosporin, cefepime, piperacillin-tazobactam quinolones, carbapenems, nitrofurantoin, trimethoprim/sulfamethoxazole, vancomycin, macrolides, metronidazole, phosphonic acids; diarrhea in the preceding week of admission; constipation, renal disease, history of hydronephrosis or pyelonephritis, vesicoureteral reflux, genitourinary abnormality other than vesicoureteral reflux, posterior urethral valve, nephrolithiasis/nephrocalcinosis, spinal muscular atrophy, west syndrome, neurogenic bladder, spina bifida, liver disease, cardiac disease, pulmonary disease, hematological disease, diabetes mellitus, lymphoma, past genitourinary surgery, femoral arterial line, radial arterial line, endotracheal tube.

The bold values in the columns corresponding to p values and confidence intervals, refer to the significant variables. The bold values in the first coloumns refer to subtitles.

However, only a history of leukemia (*p* = 0.010, AOR = 4.248, 95% CI [1.412–12.778]) and antibiotic use in the preceding 30 days (*p* = 0.012, AOR = 2.045, 95% CI [1.167–3.582]) were found as independent risk factors for UTIs caused by MDRO after adjusting for age, gender, and other potential confounders.

### Clinical presentation and initial workup

Overall, fever was the most common symptom, occurring in 81.1% of cases, followed by dysuria (31.2%), abdominal pain (29.8%), and vomiting (28.9%) (**Supplementary Figure S4**). In infants aged ≤ 2 months, primary symptoms included fever (75%) and irritability (37%). For children aged between 2 months and 2 years, additional symptoms such as vomiting and diarrhea were common. Patients aged ≥ 3 years were more likely to present with dysuria, abdominal pain, vomiting, and flank pain. Notably, enuresis was significantly more frequent in MDR cases compared to non-MDR. However, after adjusting for potential confounders, no association could be maintained.

Most of the patients were admitted with a diagnosis of UTI (50.5%) followed by fever (12.5%) (**Supplementary Table S1**). Clinical signs and symptoms on presentation, including inflammatory markers such as white blood cells count (WBC) and C-reactive protein (CRP), were similar between patients who had an MDRO and non-MDRO.

Urine samples were mostly taken by catheter (39.1%) or clean catch (25.0%). White blood cells and bacteria were detected in more than 60% and 80%, respectively, of urine analysis done on the same day of urine culture, whereas the prevalence of leukocyte esterase and nitrites was lower (49% and 41%, respectively).

### Treatment

Among patients with an MDRO, 63% were treated with a carbapenem, compared to 16% of those with a non-MDRO (*p* < 0.001, UOR = 8.786, 95% CI = [6.226–12.398]) (**Table 2**). Similarly, aminoglycosides were almost twice more likely to be administered in a case of a UTI caused by an MDRO (*p* < 0.001, UOR = 1.893, 95% CI = [1.347 - 2.660]). In contrast, third-generation cephalosporins were prescribed in 57% of UTI cases caused by non-MDRO, compared to only 13.6% of MDR cases (*p* < 0.001, UOR = 0.119, 95% CI = [0.085 - 0.166]). Regarding combination regimens, patients with an MDR uropathogen were almost four times more likely to receive two or more antibiotics, particularly a combination of an aminoglycoside with a carbapenem (*p* < 0.001, UOR = 3.994, 95% CI = [1.764 - 9.041]).

**Table 2:** Regimens used in the treatment of MDR and non-MDR urinary tract infections.

Treatment	Total	Non-MDRO	MDRO	p-value	UOR [95% CI]
*Carbapenems*	364/822 (44.3)	54/331 (16.3)	310/491 (63.1)	**<0.001**	**8.786 [6.226 - 12.398]**
*Third Generation Cephalosporins*	256/822 (31.1)	189/331 (57.1)	67/491 (13.6)	**<0.001**	**0.119 [0.085 - 0.166]**
*Aminoglycosides*	205/822 (24.9)	60/331 (18.1)	145/491 (29.5)	**<0.001**	**1.893 [1.347 - 2.660]**
*Cefepime*	65/822 (7.9)	25/331 (7.6)	40/491 (8.1)	0.757	
*Quinolones*	14/822 (1.7)	4/331 (1.2)	10/491 (2.0)	0.368	
*Piperacillin Tazobactam*	*10/822 (1.2)*	4/331 (1.2)	6/491 (1.2)	1.000*	
*TMP-SMX*	7/822 (0.9)	5/331 (1.5)	2/491 (0.4)	0.125*	
*Phosphonic acids*	1/822 (0.1)	0/331 (0.0)	1/491 (0.2)	1.000*	
***Combination of therapy***	92/822 (11.2)	17/331 (5.1)	75/491 (15.3)	**<0.001**	**3.330 [1.928 - 5.752]**
*Aminoglycosides + Carbapenems*	46/822 (5.6)	7/331 (2.1)	39/491 (7.9)	**<0.001**	**3.994 [1.764 - 9.041]**
*Aminoglycosides + Cefepime*	19/822 (2.3)	6/331 (1.8)	13/491 (2.6)	0.435	
*Carbapenems + Cefepime*	15/822 (1.8)	3/331 (0.9)	12/431 (2.4)	0.106	
*Aminoglycosides + Carbapenems + Cefepime*	12/822 (1.5)	1/331 (0.3)	11/491 (2.2)	0.053	

MDR, Multi-drug resistant; MDROs, Multi-drug-resistant organisms; TMP/SMX, trimethoprim/sulfamethoxazole.

The bold values in the coloumns corresponding to p values and confidence intervals, refer to the significant variables. The bold values in the first coloumns refer to subtitles.

### Outcome

**Table 3** summarizes the outcomes of patients in the two groups. There was no significant difference in the total duration of hospital stay or the need for intensive care unit admission. Patients with a UTI caused by an MDRO received inpatient antibiotics for a slightly longer duration (5.47 ± 3.86 days) compared to the other cohort (4.55 ± 2.96 days) (p < 0.001, UOR = 1.081, 95% CI = [1.037–1.128]).

**Table 3:** Hospital course, discharge antibiotics, and clinical outcomes in patients with UTI caused by MDRO or non-MDRO.

Hospital course and outcome	Total	Non-MDRO	MDRO	p-value	UOR [95% CI]
**Duration of hospital stay, mean days (± SD) (n = 831)**	10.84 (± 26.33)	10.46 (± 36.35)	10.80 (± 17.02)	0.853	
**NICU/PICU/SDU admission**	89/831 (10.7)	44/334 (13.2)	45/497 (9.1)	0.06	
**Duration of ICU stay, mean days** **(± SD) (n = 70)**	34.46 (± 72.52)	15.87 (± 21.50)	20.24 (± 21.81)	0.426	
**Total days of ATB, mean days (± SD)**	5.08 (± 3.56)	4.55 (± 2.96)	5.47 (± 3.86)	**<0.001**	**1.081 [1.037 - 1.128]**
**Discharge home on ATB**	411/433 (94.9)	223/226 (98.7)	188/207 (90.8)	**<0.001**	**0.133 [0.039 - 0.457]**
*Penicillin*	25/409 (6.1)	25/222 (11.3)	0/187 (0.0)	0.998	
*Second generation cephalosporins*	10/409 (2.4)	4/222 (1.8)	6/187 (3.2)	0.125	
*Third generation cephalosporins*	236/409 (57.7)	169/222 (76.1)	67/187 (35.8)	**<0.001**	**0.076 [0.028 - 0.207]**
*Cefepime*	2/409 (0.5)	2/222 (0.9)	0/187 (0.0)	0.999	
*Quinolones*	38/409 (9.3)	9/222 (4.1)	29/187 (15.5)	0.440	
*Aminoglycosides*	31/409 (7.6)	5/222 (2.3)	26/187 (13.9)	1.000	
*Carbapenem*	35/409 (8.6)	2/222 (0.9)	33/187(17.6)	0.188	
*TMP-SMX*	31/409 (7.6)	5/222 (2.3)	26/187 (13.9)	Ref	
*Cephalosporin + aminoglycoside*	1/409 (0.2)	1/222 (0.5)	0/187 (0.0)	NA	
Outcome					
*Recovered without sequelae*	681/829 (82.1)	286/333 (85.9)	395/496 (79.6)	Ref	Ref
*Recovered with sequelae*	58/829 (7.0)	23/333 (6.9)	35/496 (7.1)	0.729	
*Recurrence of infection*	78/829 (9.4)	21/333 (6.3)	57/496 (11.5)	**0.011**	**1.965 [1.165 - 3.315]**
*Death*	12/829 (1.4)	3/333 (0.9)	9/496 (1.8)	0.248	

MDROs, Multi-drug-resistant organisms; NICU, neonatal intensive care unit; PICU, pediatric intensive care unit; SDU, step down unit; ICU, intensive care unit; TMP/SMX, trimethoprim/sulfamethoxazole.

The bold values in the columns corresponding to p values and confidence intervals, refer to the significant variables. The bold values in the first coloumns refer to subtitles.

Overall, there was a high tendency to discharge patients on antibiotics (N = 411/433, 94.9%), but significantly more in non-MDR cases (p < 0.001, UOR = 0.133, 95% CI = [0.039–0.457]). This is explained by the fact that the most common antibiotic prescribed at discharge was a third-generation cephalosporin (oral or parenteral); given in 76% of patients with a UTI caused by a non-MDRO (p < 0.001, UOR = 0.076, 95% CI = [0.028–0.207]).

Most patients (85.0%) did not undergo a DMSA scan; among those who did, scarring was detected in approximately 40% of patients, irrespective of whether infection was caused by a MDRO or not. Recurrence of UTI occurred in 9.4% of patients and was twice as likely in those with a UTI caused by an MDRO (p = 0.011, UOR = 1.965, 95% CI = [1.165–3.315]).

## Discussion

Our study revealed an alarming prevalence of MDROs and ESBL-producing uropathogens in urinary tract infections among hospitalized Lebanese children, reaching 68% and 45% respectively in 2021. The literature on resistance in pediatric UTIs has primarily focused on ESBL producing organisms rather than those with concurrent non-susceptibility to three or more antimicrobial classes. Even within single-site epidemiological studies utilizing the 2010 international MDR classification (Magiorakos et al., 2012), a significant divergence in the reported percentages is observed according to geographical regions. Low rates are reported in countries from North America, Oceania, and Southern Europe, ranging between 6% to 13% (Uzodi et al., 2017; Raman et al., 2018; Esposito et al., 2021). Our results align more closely with those observed in countries from South Asia (Parajuli et al., 2017; Roul et al., 2022). A cross-sectional study conducted in India over a 1-year period detected MDROs in 64% of urine cultures of children with UTIs (Roul et al., 2022). Similarly, in Nepal, Parajuli et al. reported that 65% of 739 *E. coli* isolates were resistant to at least one antimicrobial from three different groups (Parajuli et al., 2017). Published studies on the topic from Saudi Arabia often included adults in addition to children. At a tertiary healthcare center in Riyadh, Alqasim et al. identified 67% of MDROs over a 3-month period in 2018 among hospitalized patients (Alqasim et al., 2018). Within the same year, another retrospective chart review of patients who presented to the emergency department of King Abdulaziz Medical City reported an overall MDR rate of 22.77%. However, when categorized by age, the prevalence was the highest among the elderly (50%), followed by adults (18%) and children (10.34%) (Alanazi et al., 2018).

Among the total *E. coli* and *Klebsiella* spp. isolates identified in our study, resistance ranging from 30% to 50% to oral antibiotics commonly prescribed in pediatric UTIs in Lebanon was detected. Within MDR strains, resistance was notably higher, exceeding 80% for third-generation cephalosporins and 70% for folate pathway inhibitors. This finding underscores the need to re-evaluate first line antibiotics used, as they might be ineffective empirical choices. In a previous study, we reported statistically significant linear trends in antibiotic resistance to cephalosporins and fluoroquinolones between 2001 and 2011 (Hanna-Wakim et al., 2015). In the current data, an upward trend of non-susceptibility over several years, compared to 2011, was only observed for third generation cephalosporins and folate pathway inhibitors. Our observations run parallel to the reported findings in the Arab league countries by Moghnieh et al., where resistance to third generation cephalosporins among *E. coli* and *Klebsiella* spp. exceeded 50% in some countries such as Egypt and Syria (Moghnieh et al., 2018); in contrast to the USA where recent data reveal a rate not exceeding 5% (Bradley, 2022; Dasgupta-Tsinikas et al., 2022). Trimethoprim-sulfamethoxazole resistance is high worldwide, with pooled prevalence rates reaching 30% in developed countries within the OECD. The rate is more than double in developing countries such as Lebanon and Saudi Arabia (Bryce et al., 2016; Abdelgalil et al., 2024), where a recent study reported resistance levels as high as 64%. The threat of carbapenem resistance has been emerging both in Lebanon and globally; however, its prevalence remains low, at less than 10%, consistent with our findings (Moghnieh et al., 2018; Moghnieh et al., 2019). As for aminoglycosides, up to 30% of *E. coli* and *Klebsiella* spp. were resistant to this class of antimicrobials in the current study. A comparable rate is reported in some countries of South Asia (Parajuli et al., 2017; Roul et al., 2022), whereas European data reveal resistance rates not exceeding 10% (European Centre for Disease Prevention and Control (ECDC) and the World Health Organization (WHO) Regional Office for Europe, 2023). Aminoglycosides are increasingly being used for the treatment of MDR UTIs due to multiple factors including their ability to achieve high concentrations in the urinary system and increasing data of limited nephrotoxicity (Zohar et al., 2024). More importantly, their use is driven by global efforts to spare prescribing carbapenems as much as possible to avoid associated resistance (Aksoy et al., 2022). However, our local resistance data might justify the empiric use of carbapenems rather than aminoglycosides, when suspicion of an MDRO-UTI is high, at least until the result of the urine culture is available. Of concern, 44.6% of all *E. coli* and Klebsiella spp. isolates were identified as ESBL producers over our study period, almost three times the proportion reported in our earlier study (Hanna-Wakim et al., 2015). Within MDR isolates particularly, this percentage further increased to 69.2%. This finding is expected as ESBL-producing bacteria are often MDR as well, since the plasmids they harbor frequently carry genes that provide resistance to other classes of antimicrobials, such as aminoglycosides, fluoroquinolones, and sulfonamides (Moxon and Paulus, 2016; Mahony et al., 2020). Over the 10 years of the study, statistically significant increases in the rate of ESBL producers compared to the 2011 baseline were observed in 2014, 2016, and 2021. Coinciding with the COVID-19 pandemic, we observed a decrease in ESBL prevalence from 40% in 2019 to 33% in 2020, followed by a progressive increase to 45.1% in 2021. Comparable trends were reported in Saudi Arabia where analysis of 9697 UTI patients, including adults, revealed that the likelihood of detecting ESBL production decreased by 10% during the COVID-19 pandemic (*p* = 0.040, OR= 0.91; 95% CI= 0.83–0.99) (Altamimi et al., 2024). Another study in France identified a significant monthly decrease of −0.04% to −0.22% in ESBL *E. coli* (p < 0.001) during the pandemic (Lemenand et al., 2021). This correlation could be attributed to the fact that person to person contact in the community, including schools and daycares, was limited during the pandemic. Additionally, infection control measures such as visitor restrictions, hand hygiene, and universal mask-wearing were strictly implemented; and it is well known how crucial such factors are in the epidemiology of ESBL-producing *Enterobacterales* transmission (Lemenand et al., 2021; Altamimi et al., 2024). Moreover, during complete lockdowns in 2020, healthcare utilization was largely limited to severe and urgent cases with an associated decline in antibiotic prescribing rates, partially explaining the initial drop in ESBL producing organisms (Gottesman et al., 2022). However, there was a high rate of empiric antibiotic use in COVID-19 patients, often in the absence of bacterial co-infection (Sili et al., 2024). This, combined with the lifting of mitigation measures, might have led to the subsequent rebound in ESBL rates. Independent risk factors for UTIs caused by ESBL-producing uropathogens in the literature include recurrent UTIs, neurological disorders, immunosuppressive conditions, antibiotic use within 30 days before admission, suppressive antibiotic therapy, and hospitalization in the previous month. In our study, univariate analysis showed significance of the previous factors in MDR UTIs (*p* ≤ 0.05). However, only a history of leukemia (*p* = 0.010, AOR = 4.248, 95% CI [1.412–12.778]) and antibiotic use within the preceding 30 days (*p* = 0.012, AOR = 2.045, 95% CI [1.167–3.582]) maintained significance in multivariable analysis. The link between leukemia and MDROs can be partially attributed to the fact that over 40% of the children with malignancies in our cohort were diagnosed with leukemia. Additionally, patients with leukemia require frequent hospital admissions, where MDROs are particularly common and are transmitted from others through close contact​. After this colonization, antibiotic therapy and immunosuppression from chemotherapy can disrupt the balance of the gut microbiota, promoting the overgrowth of pathogenic species. This imbalance increases the likelihood of bacterial translocation of colonizing MDR *Enterobacterales* leading to infections such as UTIs and bacteremia (Ballo et al., 2019; Duhaniuc et al., 2024).

Furthermore, as MDROs became prevalent in the community as well, transfer and colonization is increasing among immunocompetent children in the Middle East (Dandachi et al., 2019). For instance, a study conducted in Jordan found that 50% of infants were colonized with *E. coli* during the first month of life, of which 42% were MDR and 20% were resistant to carbapenems (Badran et al., 2016). In Lebanon, almost 50% of children below 1 year of age were found to be rectal carriers of ESBL *Enterobacterales* in a study by Hijazi et al (Hijazi et al., 2016). Data suggests that previous antibiotic use crucially contributes not only to MDR infections, but colonization as well. In Lebanon, the prevalence of self-medication reaches almost 50% according to the last published data (Dandachi et al., 2019). This rate is high in the Middle East in general, surpassing 80% in some countries including Jordan, Syria, and United Arab Emirates (Khalifeh et al., 2017). In contrast, European countries, where access to over-the-counter antibiotics is tightly regulated, experience much lower rates of self-medication, ranging from just 1% to 4% (Alhomoud et al., 2017). Furthermore, some studies have highlighted that the resistance induced by antibiotic overuse could persist for up to six months (Bryce et al., 2016). Our finding that children aged ≥ 5 years being nearly twice as likely to present with an MDR uropathogen could be attributed to prolonged exposure to antimicrobial agents and hence transmission from the community, as well as increased frequency of over-the-counter use of antibiotics.

The long-term implications of colonization with MDROs includes more frequent recurrence of infection, as highlighted in our data. Resistant strains often carry virulence factors such as adhesion molecules and biofilm formation capabilities that enhance their ability to persist as reservoirs in urinary tract and evade host defenses (Azam et al., 2023). Curbing the spread of multi-drug resistance requires a comprehensive multi-disciplinary approach. The most important step would be to reinforce the strict implementation of antimicrobial stewardship with strong efforts to reduce unnecessary antibiotic use in both hospitals and outpatient care (van Duin and Paterson, 2020).

### Strengths and limitations

Data from the Middle East and North Africa region relating to infections caused by MDRO remain scarce. The current study offers important insights about their prevalence and risk factors in pediatric UTI (Al Dabbagh et al., 2023). In addition, data was collected from two major tertiary care centers improving generalizability of the findings. Finally, by employing both univariate and multivariable analyses we ensured elimination of confounders and bias.

Our study has few limitations. First, its retrospective nature limits the ability to establish causal relationships and may be subject to incomplete data. Second, it is based solely on the Lebanese pediatric population, hence the findings may not be applicable to other countries. The fact that the study excluded non-hospitalized cases would underestimate the overall burden of MDROs in the community. Lastly, some variables, such as prior colonization with resistant organisms, were not assessed.

## Conclusion

This study highlights the alarming threat of MDROs among children hospitalized with a UTI in Lebanon. Risk factors such as recent antibiotic use and being a leukemia patient were strongly associated with multi-drug resistance. Although AUBMC and SGHUMC are two tertiary care centers in Lebanon where antimicrobial stewardship programs are implemented, our findings reveal that certain gaps need urgent attention. For instance, the existing programs focus on inpatient settings exclusively, although the rate of self-medication is high in Lebanon. Additionally, such programs face insufficient compliance, despite regular audits of broad spectrum antibiotic use within the hospitals, along with targeted education programs for healthcare workers. As for empiric treatment guidelines, re-evaluation is warranted, particularly considering the high resistance rate to third-generation cephalosporins, the first line antibiotics used in pediatric UTIs in both institutions. Furthermore, strict abidance to infection control measures, including isolation protocols for patients colonized or infected with MDROs, should also be reinforced.

## Data Availability

The raw data supporting the conclusions of this article will be made available by the authors, without undue reservation.
