# Supplementary material for: The challenge of multidrug resistance in hospitalized pediatric patients with urinary tract infections
Source: Front Cell Infect Microbiol. 2025 Jul 1;15:1570405. doi: 10.3389/fcimb.2025.1570405 (PMC12259619; doi:10.3389/fcimb.2025.1570405)
Supplement: Supplementary file 1 [file Table1.docx]

**Supplementary material**

**Figure 1S. Distribution of identified organisms**

**Spp.: species**

*Other organisms: Group B Streptococcus (*4), *Serratia marcescens* (*4), *Providencia stuartii* (*1), *Ghryseobacterium gleum* (*1), Salmonella (*1)

Identified species:

Klebsiella species: *Klebsiella pneumoniae*: n=100 (11.4%), *Klebsiella oxytoca*: n=11 (1.3%), Klebsiella spp. n=3 (0.3%),

Pseudomonas species: *Pseudomonas aeruginosa* n=36 (4.1%), Pseudomonas species n=1 (0.1%),

Proteus species:  *Proteus mirabilis* n=13 (1.5%), *Proteus vulgaris* n=2 (0.2%),

Enterobacter species: *Enterobacter cloacae* n=8 (0.9%), Enterobacter species n=1 (0.1%),

Enterococcus species*: Enterococcus faecium* n=4 (0.5%), *Enterococcus faecalis* n=8 (0.9%), Enterococcus species n=10(1.1%),

Citrobacter species*: Citrobacter freundii* n=5 (0.6%), *Citrobacter koseri* n=6 (0.7%),

Staphylococcus species: *Staphylococcus aureus* n=5 (0.6%), coagulase negative Staphylococcus n=4 (0.5 %)

**Figure 2S. Distribution of resistance to different antimicrobials isolates (A) *E. coli*, (B) Klebsiella spp., and (C) Pseudomonas spp.**

Antimicrobial Categories: Penicillins + beta-lactamase inhibitors (amoxicillin-clavulanic acid), antipseudomonal penicillins + betalactamase inhibitors (piperacillin-tazobactam), third generation cephalosporins (cefixime, ceftazidime, and cefotaxime), fourth generation cephalosporins (cefepime), folate pathway inhibitors (trimethoprim / sulfamethoxazole), fluoroquinolones (ciprofloxacin and levofloxacin), carbapenems (imipenem), aminoglycosides (amikacin and gentamicin).

**Figure 3S. Proportion of extended spectrum beta-lactamase (ESBL)-urinary tract infections over the study period.**

*Compared to 2011, the increase in ESBL rate was statistically significant in 2014: p≤0.001, 3.712 [1.839- 7.494], 2016: p=0.001, 3.054 [1.54- 6.058], and 2021: p=0.041, 2.236 [1.033- 4.839]

**Figure 4S. Symptoms and signs observed in the study cohort.**

MDRO: multi-drug-resistant organism

**Table 1S. Admission and laboratory characteristics of the cohort.**

|  | **Total** | **Non-MDRO** | **MDRO** |
| --- | --- | --- | --- |
| **Admission Diagnosis** |  |  |  |
| *UTI* | 419/830 (50.5) | 160/334 (47.9) | 259/496 (52.2) |
| *Pyelonephritis* | 60/830 (7.2) | 20/334 (6.0) | 40/496 (8.1) |
| *Dysuria/Urinary frequency/ Flank pain* | 19/830 (2.3) | 5/334 (1.5) | 14/334 (2.8) |
| *Fever* | 104/830 (12.5) | 53/334 (15.9) | 51/496 (10.3) |
| *Febrile neutropenia* | 28/830 (3.4) | 6/334 (1.8) | 22/496 (4.4) |
| *Bacteremia or suspected bacteremia* | 6/830 (0.7) | 3/334 (0.9) | 3/496 (0.6) |
| *Sepsis or suspected sepsis* | 17/830 (2.0) | 11/334 (3.3) | 6/334 (1.2) |
| *Gastroenteritis/Diarrhea/Vomiting/Abdominal pain/Dehydration* | 32/830 (3.9) | 13/334 (3.9) | 19/496 (3.8) |
| *Immunodeficiency or malignancy* | 23/830 (2.8) | 5/334 (1.5) | 18/496 (3.6) |
| *Chemotherapy* | 14/830 (1.7) | 5/334 (1.5) | 9/496 (1.8) |
| *Congenital heart disease* | 23/830 (2.8) | 6/334 (1.8) | 17/496 (3.4) |
| *Other diagnoses†* | 85/830 (10.2) | 47/334 (14.1) | 38/496 (7.7) |
| **WBC (n=828)** | 13,748 (±28300) | 15,066 (±16480) | 12,859 (±10655) |
| **CRP (n=617)** | 48.33 (±401.60) | 51.92 (±79.63) | 45.97 (±69.30) |
| *Radial arterial line* | 20/822 (2.4) | 10/333 (3.0) | 10/489 (2.0) |
| **ET tube** | 48/827 (5.8) | 18/333 (5.4) | 30/494 (6.1) |
| **Urinary catheter** | 70/780 (9.0) | 22/318 (6.9) | 48/462 (10.4) |
| **Method of urine collection** |  |  |  |
| *Urine bag collection* | 65/831 (7.8) | 28/334 (8.4) | 37/497 (7.4) |
| *Catheter* | 325/831 (39.1) | 141/334 (42.2) | 184/497 (37.0) |
| *Suprapubic collection* | 3/831 (0.4) | 1/334 (0.3) | 2/497 (0.4) |
| *Foley collection* | 17/831 (2.0) | 3/334 (0.9) | 14/497 (2.8) |
| *Clean catch* | 208/831 (25.0) | 82/334 (24.6) | 126/497 (25.4) |
| **Urine analysis in the same day of the positive urine culture** | |  |  |
| **WBC (n=811)** |  |  |  |
| *Negative/Rare/Few* | 284/811 (35.0) | 97/328 (29.6) | 187/483 (38.7) |
| *Numerous* | 527/811 (65.0) | 231/328 (70.4) | 296/483 (61.3) |
| **Leucocyte esterase (n=706)** |  |  |  |
| *Negative* | 133/706 (18.8) | 41/277 (14.8) | 92/429 (21.4) |
| *Slightly detected* | 227/706 (32.2) | 89/277 (32.1) | 138/429 (32.2) |
| *Strongly detected* | 346/706 (49.0) | 147/277 (53.1) | 199/429 (46.4) |
| **Nitrite (n=815)** |  |  |  |
| *Negative* | 480/815 (58.9) | 197/327 (60.2) | 283/488 (58.0) |
| *Positive* | 335/815 (41.1) | 130/327 (39.8) | 205/488 (42.0) |
| **Bacteria (n=542)** |  |  |  |
| *Negative* | 105/542 (19.4) | 36/199 (18.1) | 69/343 (20.1) |
| *Positive* | 437/542 (80.6) | 163/199 (81.9) | 274/343 (79.9) |

MDRO: multi-drug-resistant organism; UTI: urinary tract infection; WBC: white blood cells count; CRP: C-reactive protein; ET: endotracheal tube. Strongly detected refers to documentation of any of the following values: ≤500/µL, ≤3+, ≤+++; slightly detected refers to all other values,

†Other diagnoses: Prematurity, GU disease/anomalies/procedures, seizures, pneumonia, other infections, other several unrelated diagnoses
